# The Use of Diode Laser for the Surgical Removal of Pyogenic Granuloma of the Dorsum of the Tongue: A Case Report

**DOI:** 10.7759/cureus.45112

**Published:** 2023-09-12

**Authors:** Lubna M Al-Otaibi, Mohammed M Al-Ali

**Affiliations:** 1 Oral Medicine and Special Care Dentistry Department, Prince Sultan Military Medical City, Riyadh, SAU

**Keywords:** minimally invasive dentistry, oral cavity, diode laser, dorsum of the tongue, pyogenic granuloma

## Abstract

Pyogenic granuloma (PG) is a common reactive oral lesion predominantly involving the gingiva and rarely occurring on the dorsum of the tongue. It can develop at any age but more commonly in the second decade with a female predilection. Numerous factors are associated with its development, and surgical removal is the standard treatment.

Various surgical modalities have been used to excise it. Herein, we report a case of a female patient in her late 60s who presented with an exophytic lesion involving the dorsum of the tongue, which was excised using a 940 nm diode laser. In addition, it discusses the advantages of diode laser as a surgical modality and describes this lesion's clinical features and pathogenesis.

## Introduction

Pyogenic granuloma (PG) is a common reactive exophytic pedunculated or sessile soft tissue growth that can occur in the oral cavity [1,2].

The etiology of PG still needs to be clarified. However, numerous factors have been reported to be associated with its development, such as local irritation, trauma, dental plaque, hormonal factors, and some medications [2-4].

Although PG may develop at any age, the highest prevalence was in the second decade. Pyogenic granuloma occurs more commonly in females, which may be attributed to the vascular effects of female hormones [1,5,6].

The prevalence of oral pyogenic granuloma is estimated to be around 1.85% among all oral hyperplastic lesions. It primarily affects the keratinized gingiva, with a higher incidence in the maxillary gingiva than the mandibular gingiva and a greater occurrence in the anterior rather than the posterior region. Involvement of extra-gingival areas, such as the buccal and labial mucosa, palate, and tongue, is rare [7,8].

The standard treatment for oral PG includes eliminating the etiological factors and conservative surgical removal of the lesion. Various surgical modalities have been used to excise oral PG. Cryosurgery, cauterization with silver nitrate, sclerotherapy, injection of absolute ethanol, sodium tetradecyl sulfate and corticoste­roids, and neodymium-doped yttrium aluminum garnet (Nd:YAG) and CO2 laser, as well as laser photocoagulation, have all been proposed as treatment options [2,3,5,6,9].

Oral PG has no potential for infiltrative growth or malignant transformation. The recurrence rate after conservative excision is approximately 16%, with a higher chance of recurrence when it involves the gingiva or appears in pregnant women. Incomplete surgical removal, failure to eliminate the cause, and new trauma to the area can lead to recurrence [1,2,10].

Herein, we present a case of PG involving the dorsum of the tongue in a medically compromised female patient. The lesion was excised using a 940 nm diode laser, which resulted in a short healing period with no postoperative pain, ulceration, or scarring.

## Case presentation

A 69-year-old Saudi female patient presented to the oral medicine clinic in Prince Sultan Military Medical City in Saudi Arabia with a chief complaint of a lesion on her tongue. The patient had noticed this lesion about one year ago. It was painless and did not increase in size.

The patient was previously diagnosed with hypertension, hyperlipidemia, osteoarthritis, and osteoporosis. She was under medical care for these conditions and was on the following medications: atenolol, atorvastatin, and IV denosumab. The extraoral examination was unremarkable.

The intraoral examination revealed a painless, well-circumscribed, exophytic, sessile, red, nodular lesion with a pink base (approximately 2 mm in depth) located on the right side of the dorsum of the tongue. It was rubbery in consistency and measured about 0.5 × 0.6 cm. Our clinical differential diagnosis included pyogenic granuloma, hemangioma, vascular malformation, and foreign body granulomatosis. Differentiation between these lesions depending only on the clinical appearance is impossible. Thus, biopsy and histopathological assessment of the obtained biopsy are required to reach the final diagnosis.

**Figure 1:**
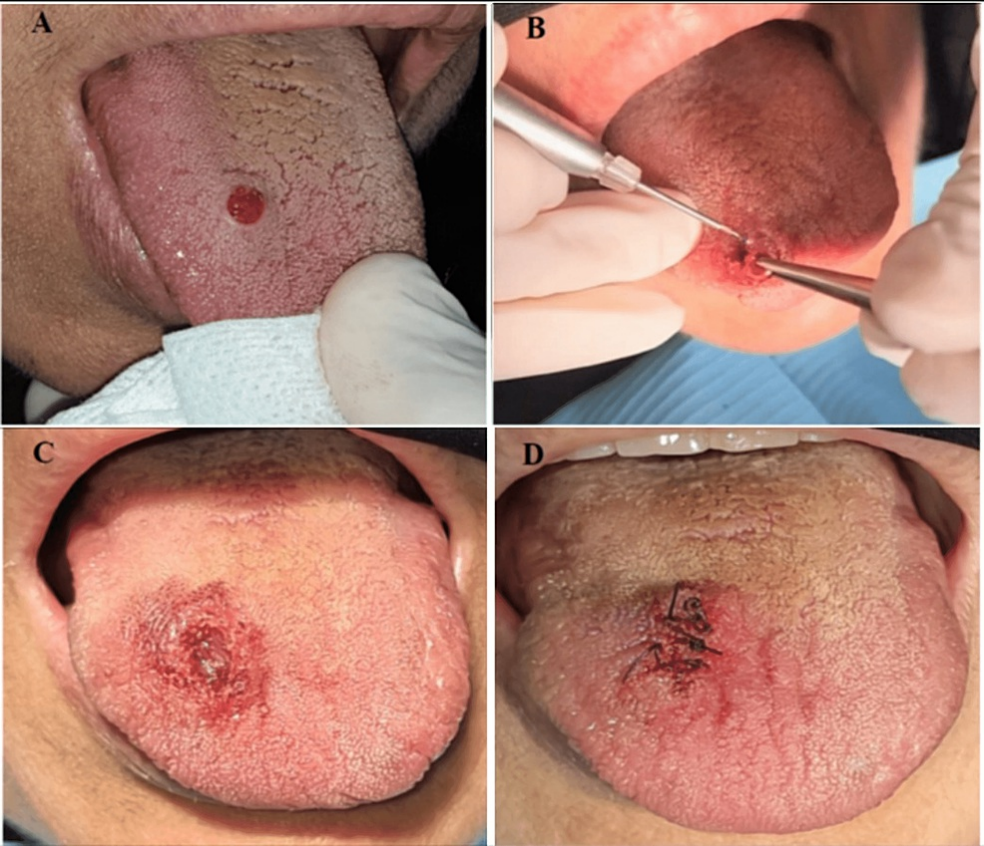
Intraoral photographs A: Preoperative photograph showing an exophytic, sessile, red, nodular mass with a pink base (0.5 × 0.6 cm). B: Excision of the lesion using a 940 nm diode laser (E4-4mm surgical tip). C: The surgical site immediately after excision and hemostasis achievement. D: The wound was closed using 4/0 coated VICRYL sutures.

Patient laboratory tests were within normal limits, including complete blood count with differential, blood coagulopathy, and general profile. The lesion was excised under local anesthesia (mepivacaine 2%, approximately 1 mL) using a 940 nm diode laser (Epic Biolase, Irvine, CA, USA) with an initiated E4-4mm surgical tip. The tip was applied in contact mode with a focused beam using the circumferential incision technique to excise the tissue (3.5 W, continuous wave mode). Bleeding was then stopped using the hemostasis setting (0.5 W, continuous wave mode), and two interrupted 4/0 coated VICRYL sutures were placed to avoid food and debris entrapment and contamination of the surgical site (Figure 1A-1D). No postoperative medications were prescribed.

**Figure 2:**
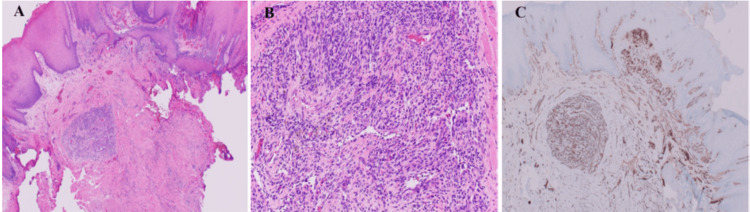
Hematoxylin and eosin-stained and IHC microscopic photographs of the lesion A: Low magnification shows fibrous connective tissue stroma with variable shaped and sized blood vessels with a central lobulated area (×4). B: Higher magnification of the lobulated area shows a highly vascular proliferation of granulation tissue and endothelial cells (×20). C: CD31 IHC stain highlights blood vessels and stained endothelial cells. IHC: immunohistochemistry, CD31: cluster of differentiation 31

The specimen was placed in 10% neutral buffered formalin and was sent for histopathological examination. Microscopic examination showed a parakeratinized stratified squamous surface epithelium. The underlying connective tissue stroma was fibrous with numerous and various shaped and sized engorged blood vessels with a central lobulated area of a highly vascular proliferation of granulation tissue and endothelial cells. The endothelial cells were positive for CD31 (Clone JC70, Ventana/Roche, Tucson, AZ, USA) and negative for human herpesvirus 8 (HHV8) and cytokeratin (CK) AE1/AE3. Accordingly, the diagnosis of pyogenic granuloma (lobulated capillary hemangioma) was reached (Figure 2A-2C).

**Figure 3:**
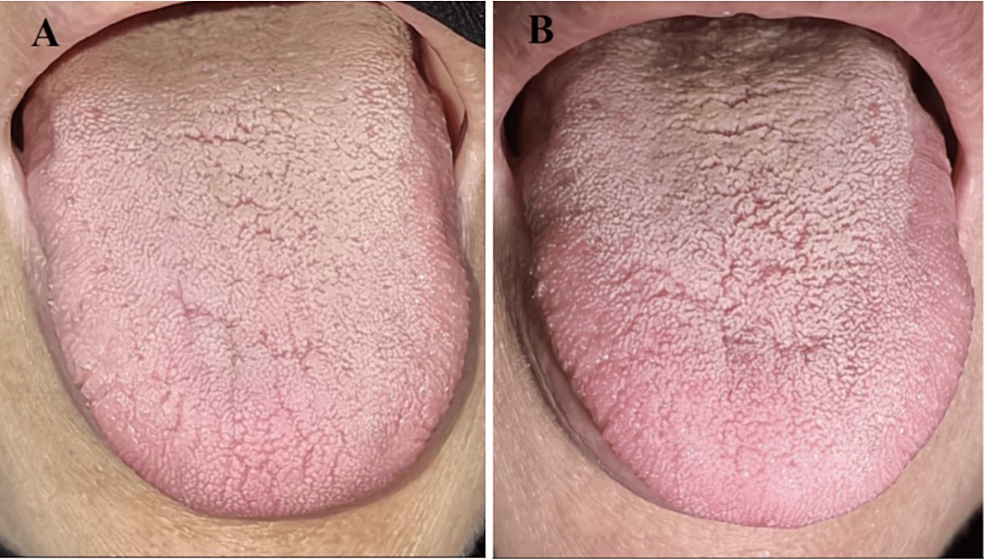
Intraoral photographs of the operation site A: Two weeks after the surgery. B: Three months after the surgery.

The patient reported no postoperative discomfort, pain, or complication. The lesion showed adequate healing after only two days post-surgery. The two-week follow-up visit observed complete healing without ulceration or scarring. The patient was seen again at a three-month post-surgery evaluation visit and was found to be doing well with no complaints or recurrence of the lesion (Figure 3A, 3B).

## Discussion

Pyogenic granuloma is a reactive hyperplastic neoplasm involving the oral cavity. Some authors claimed that the condition was initially reported by Hullihen in 1844, and the term pyogenic granuloma was established by Hertzel in 1904. Others claimed that Poncet and Dor originally reported the condition in 1897 as botryomycosis hominis, as they had suspected a fungal cause, while Crocker later introduced the term pyogenic granuloma in 1903 [2,3,5,11]. Hence, disagreement in the literature concerning PG's first report and nomenclature exists.

Various terms have been used in the literature for PG, such as granu­loma pyogenicum, eruptive hemangioma, and granulo­ma gravidarum. The term pyogenic granuloma has also been considered inaccurate and misleading since this lesion is not accompanied by pus nor histologically resembles granuloma [12,13].

Clinically, PG usually manifests as a solitary, soft, exophytic, painless, pinkish-to-red mass with a pedunculated or sessile base that can be easily injured, causing bleeding [3,14]. However, in our clinical case, it was pinkish to red in color with a rubbery base. This may be due to the fact that it was a long-standing lesion in an easily injured location.

The highest prevalence of PG occurs in the second decade of life, with the gingiva being the most commonly involved intraoral site [1,5,6]. In our case, the lesion developed in an elderly female and on the dorsum of the tongue, which is considered rare.

The etiology of PG remains unclear, and numerous factors are associated with its development [2-4]. In this presented case, given the location of the lesion, trauma was suspected to have caused its development. However, the patient consistently denied any history of trauma or tongue-biting habit.

The clinical manifestation of PG can mimic other angiomatous intraoral lesions, presenting a significant obstacle to clinical diagnosis [15]. The differential diagnosis of PG involving the dorsum of the tongue includes hemangioma, vascular malformation, Kaposi sarcoma, angiosarcoma, and non-Hodgkin lymphoma.

The standard treatment of oral PG is conservative surgical removal and elimination of etiological factors. Various surgical modalities have been used to excise oral PG [2,3,5,6,9]. During conventional scalpel blade surgery, PG may bleed easily and abundantly due to its high vascularity. This may restrict the surgeon's visibility, increase the operation time, and increase the chance of edema, hematoma, and postoperative pain [16,17]. Many advantages of using the diode laser compared to the scalpel blade have been discussed in the literature. These include decreasing the need for local anesthesia; hemostatic properties causing less intraoperative bleeding and need for sutures; shorter operation and healing time; microbial inhibition and destruction, thus maintaining a sterile condition; and reduction of postoperative pain and complications [17-20]. However, none of these studies included laser excision of a PG located on the tongue. Thus, to the best of our knowledge, this case represents the first to be reported.

In this reported case, where a 940 nm diode laser was used for the surgical excision, the procedure was found to be simple to perform with minimum bleeding and excellent precision. It was well accepted by the patient, who showed no signs of fear or discomfort during the procedure. She also reported no postoperative pain or complications. Optimum healing was achieved with no residual ulceration or scarring within two weeks. Moreover, the excised specimen was adequate for histopathological examination.

## Conclusions

We presented a case of pyogenic granuloma, which was surgically removed using a 940 nm diode laser. The procedure was accomplished with minimum bleeding and discomfort. Postoperative healing occurred rapidly and without postoperative pain or scarring.

Diode-assisted surgical excision provides a stress-free environment with a more comfortable experience for both the clinician and patient; thus, it should be considered as a practical alternative to the traditional surgical technique.
